# Improving Localization Accuracy of Neural Sources by Pre-processing: Demonstration With Infant MEG Data

**DOI:** 10.3389/fneur.2022.827529

**Published:** 2022-03-23

**Authors:** Maggie D. Clarke, Eric Larson, Erica R. Peterson, Daniel R. McCloy, Alexis N. Bosseler, Samu Taulu

**Affiliations:** ^1^Institute for Learning and Brain Sciences, University of Washington, Seattle, WA, United States; ^2^Department of Physics, University of Washington, Seattle, WA, United States

**Keywords:** magnetoencephalography (MEG), artifact, movement compensation, infant, signal space separation, brain, signal space projection, signal processing

## Abstract

We discuss specific challenges and solutions in infant MEG, which is one of the most technically challenging areas of MEG studies. Our results can be generalized to a variety of challenging scenarios for MEG data acquisition, including clinical settings. We cover a wide range of steps in pre-processing, including movement compensation, suppression of magnetic interference from sources inside and outside the magnetically shielded room, suppression of specific physiological artifact components such as cardiac artifacts. In the assessment of the outcome of the pre-processing algorithms, we focus on comparing signal representation before and after pre-processing and discuss the importance of the different components of the main processing steps. We discuss the importance of taking the noise covariance structure into account in inverse modeling and present the proper treatment of the noise covariance matrix to accurately reflect the processing that was applied to the data. Using example cases, we investigate the level of source localization error before and after processing. One of our main findings is that statistical metrics of source reconstruction may erroneously indicate that the results are reliable even in cases where the data are severely distorted by head movements. As a consequence, we stress the importance of proper signal processing in infant MEG.

## Introduction

Magnetoencephalography (MEG) is a functional imaging technique that offers excellent temporal resolution and good spatial resolution. The sensors in the MEG helmet measure the weak magnetic fields associated with electrical currents produced in the brain, e.g., during sensory, motor or cognitive tasks. The spatial sources of the detected magnetic fields can be estimated using a combination of anatomical information (digitized head shape, structural MRI) and known properties of electromagnetic field propagation, a process known as “source localization.” MEG is also passive, silent, and non-invasive, making it an excellent tool to study neural dynamics in the developing brain. However, MEG is known to be extremely sensitive to artifacts and distortions that can affect source localization. In adult populations, some artifacts can be minimized by, e.g., asking participants to stay still during the measurement, which reduces signal distortions caused by head movements. Such approaches fail in measurement sessions with awake infant subjects, and, therefore, efficient signal processing methods for movement compensation are essential for reliable infant MEG analysis. Additionally, other infant-specific distortion mechanisms exist, and based on our experience, the three most significant issues in infant MEG data that bias source localization are: (1) frequent head movement; (2) decreased signal-to-noise ratio (SNR) from increased scalp-to-sensor distance; and (3) strong cardiac artifacts which resemble brain signals in their spatial distribution. The SNR issue is mainly related to the relative positioning of the MEG sensor array and the infant head, and in this paper we focus on the methodology concerning points (1) and (3) above.

A number of processing methods have been developed to address the issues listed above, many of which exist in both adult and infant data. For example, head movement, its effect on MEG data and subsequent results after the application of movement compensation has been shown in adults (1), school-aged children (2), infants (3), as well as in clinical populations (4). In this paper, we review some of the most relevant methodological aspects of processing and analysis of infant MEG data. Special emphasis is given to the infant-specific mechanisms of signal distortion leading to source localization errors. Using real movement information from a set of 6, 7, and 12-month-old subjects, we show the effects of these distortions on magnetic field topographies and source localization, using several different processing approaches. Notably, we demonstrate that statistical metrics, such as the goodness-of-fit of equivalent current dipole models, do not necessarily capture source localization bias, meaning that significant source localization errors may remain undetected in data that have not been processed with movement compensation algorithms.

## Signal Distortions and Their Correction in Infant MEG

### External Artifacts

External artifacts arise from generators of magnetic fields that lie outside the body of the MEG subject. Common sources are power lines, elevators, electronic devices, moving vehicles, and mechanic vibration of the room housing the MEG instrument. Signal space separation (SSS) (5, 6) and its temporal extension, temporal signal space separation (tSSS) (7, 8) are commonly used methods that compensate for external interference artifacts in MEG data. The signal space separation method (SSS) is based on Maxwell's equations, where spatially discretized samples of magnetic flux (MEG data) are decomposed into amplitude coefficients of basis functions that span detectable magnetic fields in space that is free of sources of magnetic fields, i.e., in the region where MEG sensors are located. Since separate linearly independent basis functions exist for signals generated inside and outside the sensor volume, SSS provides a straightforward means of removing field components attributable to external sources. In cases where the artifact sources are not clearly distinguishable as internal or external, the temporal extension of SSS (tSSS) can be used to detect and remove components arising from these nearby artifact sources. Other efficient and widely used interference suppression methods include, e.g., signal space projection (SSP) (9) and reference sensor-based methods (10). For a review of MEG artifacts and their suppression, see Taulu et al. (11).

### Physiological Artifacts

MEG is also sensitive to physiological artifacts which arise from generators inside the body of the subject (e.g., heart, eyes, skeletal muscles). Blink- and saccade-related artifacts tend to occur less frequently in infants than adults, as the mean spontaneous blink rate in infants is <2 blinks per minute (12). In contrast, infant and child cardiac artifacts are often more than an order of magnitude larger than the brain signals of interest (13) and appear as volume currents within the skull due to the shorter distance between the heart and the MEG sensors. Additionally, infant heart rate is much faster than adults: from newborn to 6 months of age the mean heart rate is 125–145 beats per minute (bpm), compared to a mean heart rate of 80 bpm in adolescents and adults (14). Furthermore, the QRS peak is narrower for infants than for adults, ranging from 50 to 80 ms in duration, contributing electromagnetic activity in the 12.5 to 20 Hz range (14). Adolescents, on the other hand, have a QRS complex lasting between 90 and 110 ms (15). Both the increased proximity and frequency of magnetic contributions from the heart make it essential to properly characterize and remove cardiac artifacts in infant data.

Common methods used to remove both ocular and cardiac artifacts in MEG data include independent component analysis (ICA) (16–23) and SSP based on principal component analysis (PCA).

### Movement Related Artifacts

Head movements are generally unavoidable in infant populations (3), due to the larger space available for head movements (in common adult sized helmets) and infants' inability to remain still on command. Such movements distort the magnetic field distribution across the MEG sensors and can result in large errors in source localization (1). Fortunately, movement compensation algorithms have been developed to repair such artifacts (6, 24). Using head position indicator (HPI) coils that emit high-frequency sinusoidal fields, the head position can be continuously and accurately determined during an MEG recording. By forcing the spatial “expansion origin” of the internal basis functions derived from SSS to match the origin of the head coordinate system (even as the head moves over time), one can decompose the MEG signals into a representation that is specific to the brain regardless of its location with respect to the sensors. Thus, by continuously tracking the head position, the basis function coefficients can be interpreted in a static head coordinate system, as if the subject had remained still. Typically, the coefficients are used to create a virtual sensor-level signal representation corresponding to some target head position defined by the user. This is the basis of head movement compensation, and it is an essential task in the processing of infant data as will be demonstrated in subsequent sections.

### Effect of Noise Covariance

Sensor covariance matrices, which quantify the spatial correlation structure between each pair of sensors, are central to many MEG source localization algorithms such as minimum norm estimation, equivalent current dipole fits, mixed-norm solvers, and beamformers, as well as many applications of machine learning to MEG data. Covariance matrices are typically estimated from the data, either from specific segments during a subject recording (e.g., the baseline period before each trial as a “noise covariance,” or during the trial for a “data covariance”) or from data recorded just before or after the experimental session (“empty-room” data). It is important that the true underlying sensor covariance structure is accurately reflected in the estimated noise covariances, otherwise a reduction in SNR and errors in source localization can be introduced (25). In the context of source localization of movement-compensated data in particular, it is also important that full (rather than diagonal) noise covariances are used (3).

Fortunately, direct empirical source covariance estimates can be improved by using automated regularization techniques (26). However, even when using such techniques, it is important to properly account for the rank of the data. In source imaging for example, the pseudo-inverse square root of the noise covariance matrix must be computed to whiten the data. During this computation, the rank of the data must be explicitly accounted for in order to avoid amplifying data components that are numerical noise. Common operations such as SSP, ICA, and SSS can all reduce the rank of the data, and this must be explicitly taken into account (26). In other words, the noise covariance rank (and effective null space) directly affect the accuracy of source localization (and by extension, other methods that rely on covariance estimates).

In the context of movement compensation, the data rank must be taken into account carefully. The number of components used to reconstruct data can vary as a function of time, as the different head positions can yield different regularized internal bases in MNE-Python's implementation of Maxwell filtering. While a sensor covariance computed from the movement-compensated data can directly reflect this, empty-room data processed directly using SSS by default will not—it will reflect the rank of non-movement-compensated data (i.e., as if the head remained stationary), which will likely differ. Therefore, it is important that empty room data are processed the same way as movement compensated data, i.e., by using the same initial device-to-head transformation, expansion origin (in the head coordinate frame), and time-varying head position parameters as the actual data, despite the fact that there was no actual subject motion during the empty room recording.

### Reduced SNR of Infant MEG Measurements

Magnetic fields from any source (including sources in the brain) decay rapidly with distance. In traditional adult superconducting quantum interference device (SQUID)-based MEG systems, helmets are designed to place the sensors as close as possible to the helmet's inner surface, given the restrictions posed by thermal insulation between the head and the liquid helium vessel containing the SQUID sensors. When this larger helmet is used with infants, the distance reduces the strength of the measured magnetic field and negatively affects the SNR. At the same time, infants' smaller heads allow for a considerable range of movement inside adult-sized helmets. In SQUID systems, the sensors are not attached to the head, and therefore movement of the head relative to the sensors significantly distorts the distribution of the magnetic signals and thus biases source localization. While this effect is not an artifact in the strict sense, the resulting reduced strength of magnetic field components originating from the brain makes the suppression of external and physiological artifacts all the more critical. In the rest of this paper, we illustrate some of the above-mentioned artifact suppression approaches and demonstrate some common pitfalls when processing infant MEG data.

## Methods

To quantify the effects of SSS, movement compensation, and noise covariance on source localization of infant MEG data, we simulated data based on real infant head movements. Simulated datasets were analyzed using four different methods: (1) no artifact suppression (Raw), (2) Maxwell filtering (SSS), and (3, 4) Maxwell filtering with movement compensation using two different covariance estimation methods (see Data Processing, below). In addition to simulated data (where ground truth source locations are known), we show the effect of each approach on real infant data to illustrate what the effects look like in practice.

### Subjects and Data Acquisition

Data from nine 6-month, twenty-three 7-month, and fourteen 12-month-old typically developing subjects were drawn from two previously conducted studies at the University of Washington Institute for Learning and Brain Sciences. All infants were from monolingual English-speaking environments, had no reported hearing difficulties, no history of ear infections, and were born full-term (between 39 and 42 weeks of gestational age). Both studies were in accordance with the ethical standards of the institutional and/or national research committee and with the 1964 Helsinki declaration and its later amendments or comparable ethical standards. Informed consent was obtained from parents or caregivers of all infants included in both studies. MEG data were recorded in a magnetically shielded room with a whole head adult-sized 306 channel Elekta Neuromag® MEG system (Elekta Oy, Helsinki, Finland). Prior to scanning, each subject had a fabric cap fitted to the head, with five (83,143, 203, 263, 323 Hz) HPI coils attached. Anatomical landmarks (left and right preauricular points, nasion), HPI coils and additional points along the head surface were digitized using Fastrak® 3D digitizer (Polhemus, Colchester, VT, USA) to construct an individual Cartesian head-centric coordinate system. Infants were seated in a custom-made chair under the MEG helmet while listening to various auditory stimuli. For specific details about the paradigm, see Kuhl et al. (27) and Mittag et al. (28).

### Data Processing

MEG data were pre-processed using MNE-Python (29, 30). All data were analyzed using four different methods: (1) no artifact suppression (Raw), (2) Maxwell filtering only (tSSS for experimental data and SSS for simulated data), and (3, 4) two versions of Maxwell filtering with movement compensation and translation to the time-averaged head position: one localized using a covariance from (simulated) empty-room data processed using plain SSS (MC_erm−cov_); and one using a noise covariance computed from the baseline of the simulated data (MC). Note that, based on how the simulations were set up, a noise covariance calculated from simulated empty-room data that had been processed using the same time-varying position parameters as the simulated data would be equal to the baseline covariance computed from the actual data, and hence the difference between (3) and (4) tells us the importance of processing empty-room data using the same time-varying position information as task data.

For data processed with SSS, an internal expansion order of 6 and an external order of 3 was used. The internal order is smaller than the default of 8, which is typically used in adult measurements, and it is justified by the fact that the infant head is smaller and the overall source-to-sensor distance tends to be larger than in adult subjects. Data processed with movement compensation were transformed to the mean of each individual's head positions. To examine the effect of cardiac artifacts, PCA was used to identify cardiac artifacts from ECG electrodes. Signal space projection (SSP) was used to suppress the cardiac signal in the MEG data by estimating two orthogonal vectors capturing the spatial structure of heartbeats.

### Data Simulations

For each subject, real subject time-varying head movements were applied to the simulated brain sources to yield simulated sensor data that mimicked the movement distortions seen in real recordings. Source space activations were constructed by fitting a sphere to the points along the head surface which were collected during the digitization process. The sphere was used to create a volumetric grid in which sources with random orientations were simulated along 2 cm spacing at least 10° away from radial orientations relative to the center of the sphere as in Larson and Taulu (3). The dipole spacing was fixed across subjects, but due to differences in head sizes, the number of dipoles differed across subjects, averaging (mean ± 1 SD) of 88.2 ± 13.7, 93.3 ± 7.6, and 112.3 ± 15.3 for the 6, 7, and 12 month groups, respectively.

The source time course for each subject was constructed by individually activating a 100-nAm peak single source every 50 ms. In addition to these activations, the source time course included a (−200, 0) ms baseline period with no simulated brain activity, to be used in the noise covariance estimation. In addition, Gaussian noise was added to the sensors. For additional details see (3). Each of the simulated datasets were then analyzed using the four different methods: Raw, SSS, MCerm-cov, and MC.

### Fitting of Equivalent Current Dipoles

To investigate the accuracy and reliability of source localization, we fit equivalent current dipoles (ECD) to the simulated data. This procedure entails choosing the location, orientation, and strength of current dipoles so as to reconstruct the data as accurately as possible. Mathematically, a non-linear optimization algorithm searches for the best ECD parameters until the goodness-of-fit (GOF) value is maximized. Expressing the measured or simulated whitened data vector and the modeled whitened data vector as **d** and **m**, respectively, the GOF value is given as GOF = 1–(**d-m**)^T^(**d-m**)/**d**^T^**d**, where T indicates transpose. Thus, the numerical value of GOF is in the range 0...1 (0...100% fit). We define the localization error as *e* = |**r**_q_-**r**_e_|, where **r**_q_ and **r**_e_ are the true and estimated ECD location, respectively.

## Results And Recommendations

### Compensation for Movement-Related Field Distortions

**Figure 1:**
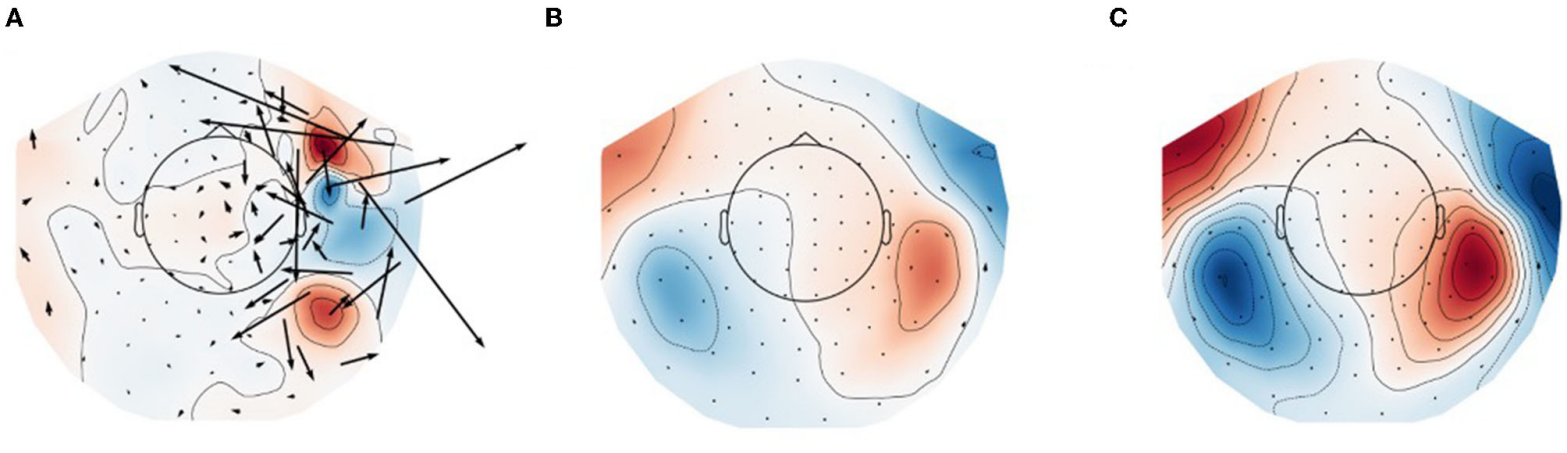
Field topographies of bilateral evoked auditory responses (at 150 ms) in a 6-month-old participant. **(A)** Raw data (no artifact suppression), **(B)** data processed with tSSS, and **(C)** data processed with tSSS + movement compensation. Black arrows represent estimation of equivalent current flow at the MEG sensor locations.

Figure 1 shows field topographies of real data from a 6-month-old subject from Mittag et al. (28), averaged across trials with an auditory stimulus, processed in three different ways. In Figure 1A no compensation is done for subject movement, and the magnetic field topography of the evoked response is clearly adversely affected by external artifacts and subject movement. After the application of tSSS to the raw data (Figure 1B), the field pattern resembles comparable adult data, and after application of movement compensation (Figure 1C) the spatial details of the modeled field are further improved. In particular, the movement-induced smooth appearance of the topography is compensated for in Figure 1C as compared to Figure 1B.

### Source Localization Error

**Figure 2:**
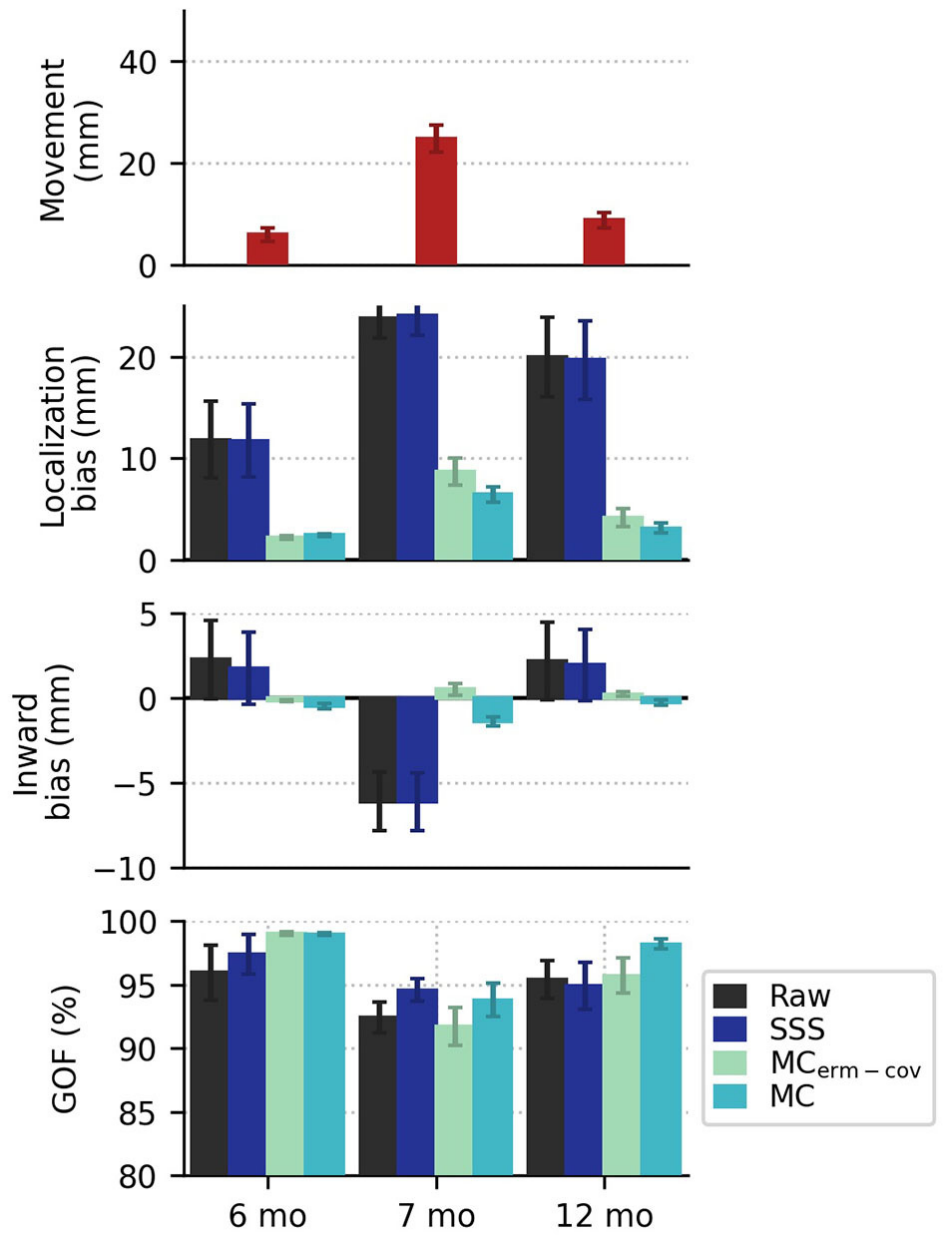
In the top row, the mean plus or minus standard error (across subjects) of the position deviation (from each subject's mean head position) shows smaller movements in the more recent recording (published in 2021) compared to an older recording (published in 2014). In the second row, localization bias is reduced by movement compensation, with greater reduction in bias when the covariance is computed from movement-compensated data. There are systematic outward and inward (relative to the head center) localization biases shown for the newer (2021) and older (2014) data, respectively. In the last row, mean goodness of fit values exceed 80% in all cases.

Using simulated data distorted by head movements from real recordings, we analyzed localization bias and goodness-of-fit under the four different processing strategies described in Methods: Data Processing. The head movements were drawn from Kuhl et al. (27) (7 mo) & Mittag et al. (28) (6 and 12 mo). The top panel of Figure 2 shows that between the first experiment (27) and the second experiment (28), there was an improvement in subject compliance in terms of reduced movement, as reflected in less subject deviation from the mean head position. For the 6, 7, and 12 month groups, paired *t*-tests of the bias of the two movement compensation modes for each group were *p* = 0.1100, 0.0061, and 0.0334, respectively. Nevertheless, even the smaller head movements seen in the later study yield localization biases in excess of 10 mm when movement compensation is not applied to the data (Figure 2, second panel). Notably, if we source localize using a noise covariance from empty-room data processed with plain SSS (without applying equivalent movement compensation to the empty-room recording), we find that source localization is adversely affected, most noticeably in the data simulated from 7-month-old's head movements. In all cases, acceptable goodness-of-fit values are obtained (>80% in all cases), even when mean localization bias exceeds 20 mm. In some cases the GOF values are actually higher in the Raw and SSS conditions compared to the movement-compensated conditions, showing that high GOF does not necessarily indicate high localization accuracy.

**Figure 3:**
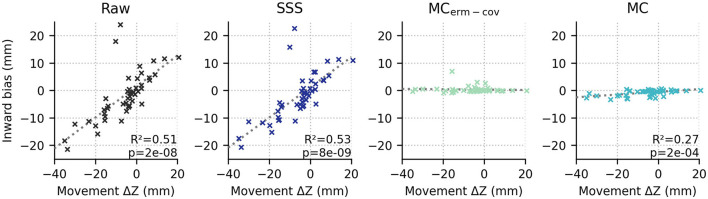
The average position in device coordinates relative to the initial position (x axis; +Z means upward) is strongly correlated with the inward bias of the ECD fit dipoles relative to the true source locations for raw and SSS-processed data (first and second columns), but not either movement-compensated case (*p* > 0.05, both).

Looking at systematic effects observed in the localization bias, we see that inward bias (as quantified by the radius of the true source minus the radius of the ECD fit location, relative to the head center) for raw and SSS-processed data is positive for 6 and 12 months, and negative for 7 months (see Figure 3). If the subject-by-subject inward bias is compared to their average upward movement (+Z in MEG device coordinates), a very strong Pearson correlation is observed for both Raw (*R*^2^ = 0.51, *p* = 1e-8) and SSS-processed data (*R*^2^ = 0.54, *p* = 5e-9), suggesting that subject head deviation from the initial position upward or downward in the MEG helmet tends to manifest as inward and outward source localization bias, respectively.

### Suppression of Cardiac Artifacts

**Figure 4:**
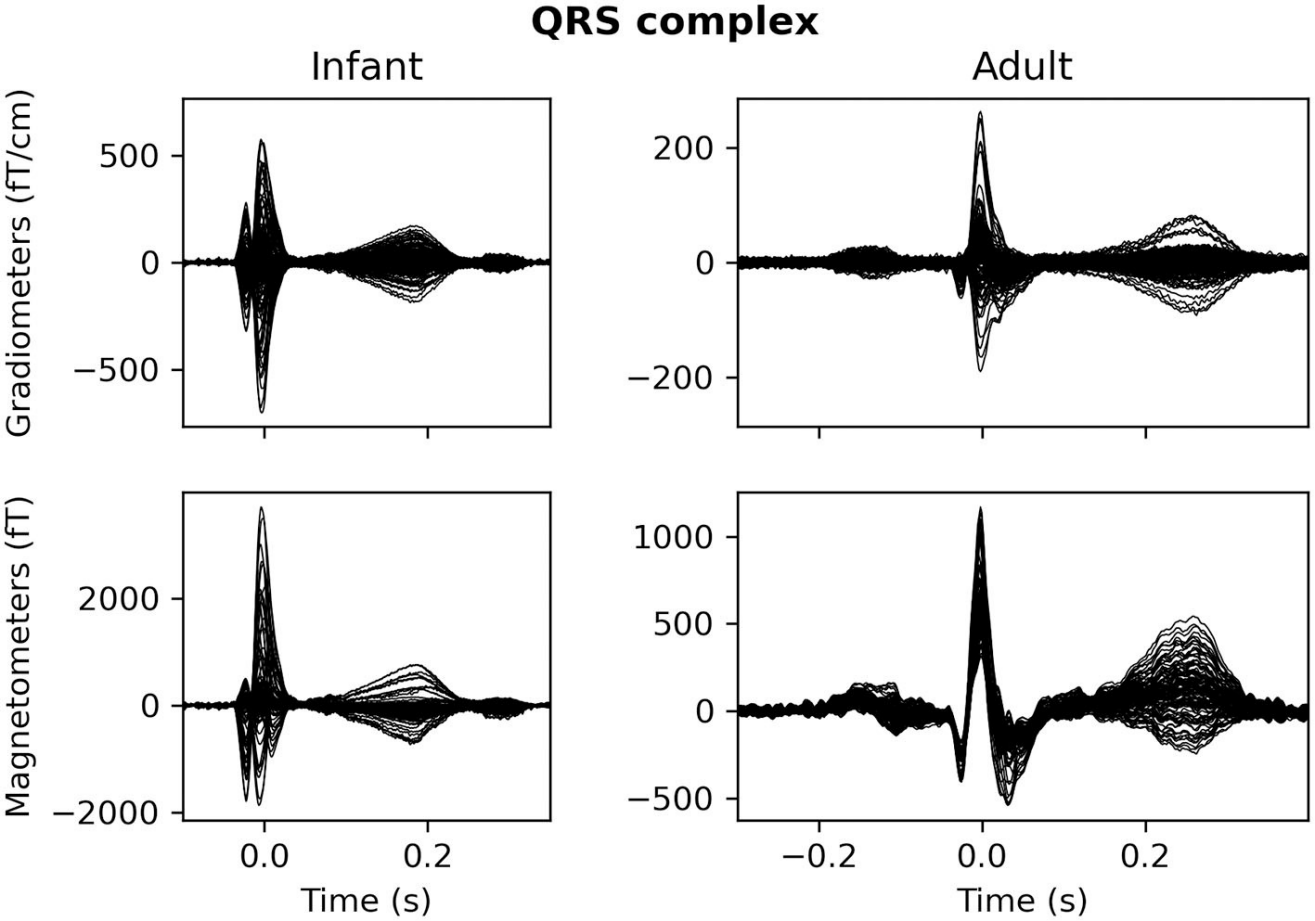
The average QRS complex from exemplar infant **(left)** and adult **(right)** MEG recordings.

As mentioned above, infant heart rates tend to be much higher compared to adolescent or adult heart rates, and the QRS complex of infant heart artifacts has a shorter duration as well (Figure 4).

**Figure 5:**
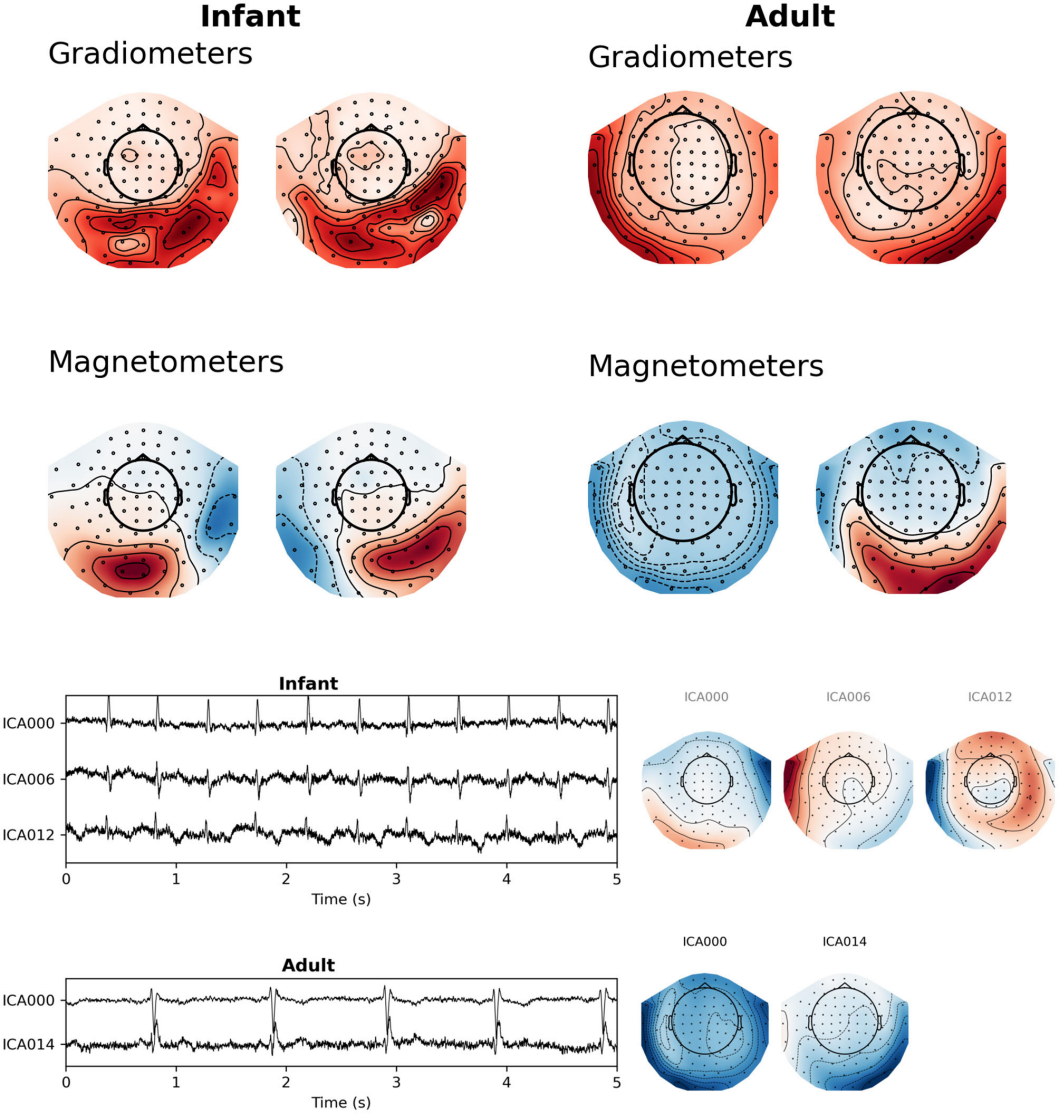
Topographic maps of cardiac fields for sample infant and adult MEG recordings. **(Top)** SSP projector fields computed from a 100 ms window surrounding the average QRS peak from this figure was sufficient to capture the artifact. **(Bottom)** latent sources and field maps for cardiac-related latent components computed with ICA. Both approaches show that fields from heart-generated volume currents in the brain may appear shallow or asymmetrical compared to adult cardiac fields.

**Figure 6:**
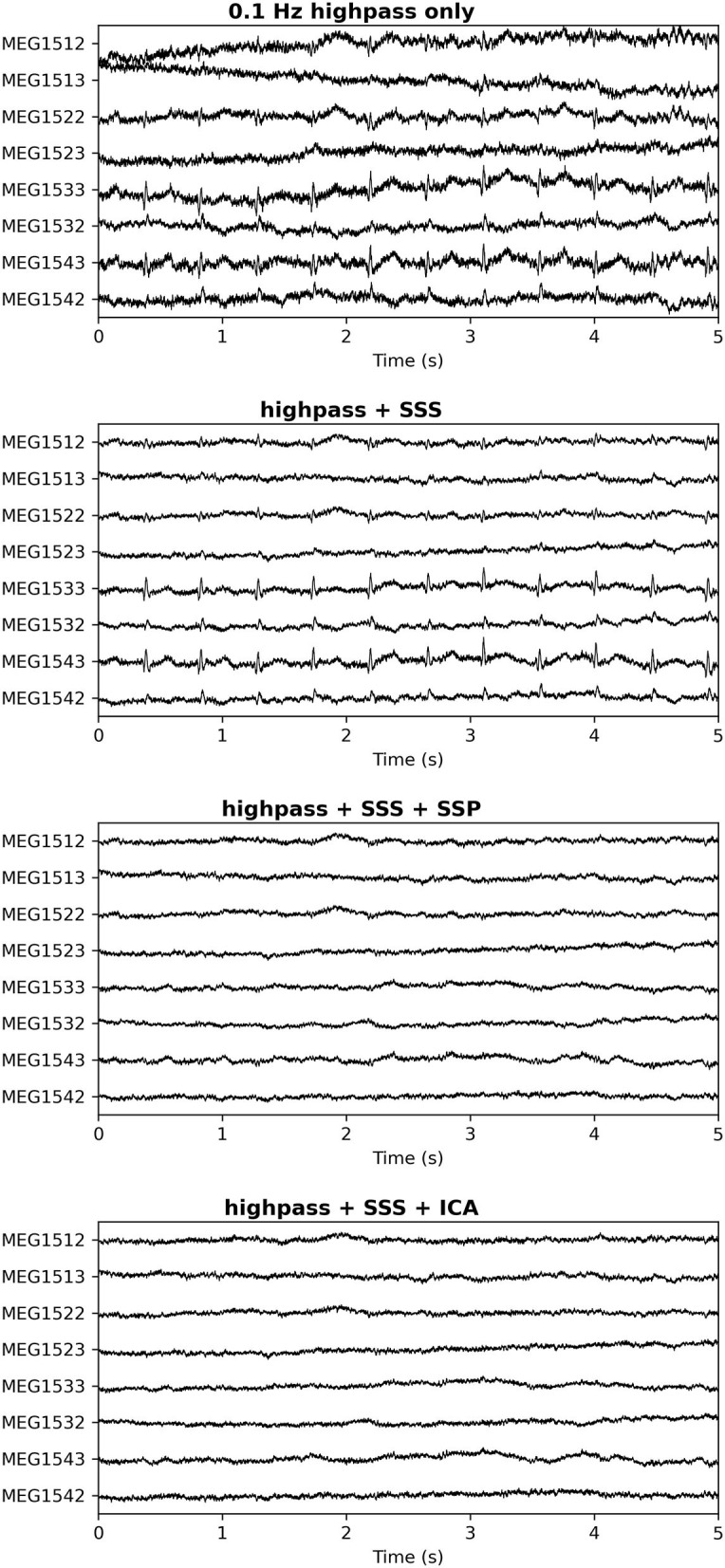
Cardiac artifact on a subset of channels after different pre-processing approaches. Top: No processing; Second Panel: SSS only; Third Panel: SSS followed by SSP (2 orthogonal projectors); Bottom Panel: SSS followed by ICA (4 cardiac-related latent sources removed). In the third and bottom panels the cardiac artifacts have been successfully repaired. The spatial distribution of the cardiac artifact stays virtually intact in the SSS process, indicating that most of its signal energy comes from the internal SSS volume where the head is located.

Additionally, in our experience a characteristic difference between cardiac artifacts in infants and adults is the fact that in infants, the spatial field distribution of the cardiac artifact often tends to be very similar to a plausible brain signal (Figure 5), which makes its algorithmic suppression inherently important and difficult. This observation is confirmed by the fact that in many cases SSS reconstruction leaves the cardiac artifact intact, cf. Figure 6.

Generally speaking, it is possible to overcome these challenges by choosing among different artifact suppression methods. For example, ICA may be more successful than SSP for some infant datasets, and both are likely to be more successful than SSS alone (though SSS is still useful for suppression of external artifacts, and can be used alongside SSP or ICA; cf. Figure 6).

## Discussion

In this paper, we have reviewed the most important challenges that may distort infant MEG data and thereby cause bias to the associated source estimates. The topics covered are based on our extensive experience with awake infant subjects and many of these aspects are relevant to clinical MEG as well, e.g., in epilepsy studies (4) or other settings where patients may have difficulties staying still during the recording. We also reviewed some of the most efficient processing methods that can correct for these distortions along with results that demonstrate the processing results and the associated accuracy of source localization (see Figure 7; a schematic of data processing in the Supplementary Material). Most importantly, we demonstrated that without application of movement compensation the source localization accuracy in infant MEG is severely compromised, with localization errors >20 mm in many cases, while statistical metrics such as the goodness-of-fit erroneously indicate high reliability of the obtained estimates based on non-compensated data. However, even with large head movements, the SSS-based movement compensation method is efficient, significantly reducing source localization bias to a few millimeters while the GOF value of source localization is almost intact compared to the uncompensated data. Consequently, one cannot solely rely on statistical confidence metrics of source modeling methods in the case of MEG signals that have been distorted by head movements. The reason is that the topography of the MEG signal distribution may resemble the pattern of a plausible brain signal despite movement-induced spatial modulation, which has to be compensated for in order to obtain robust interpretation of the data. This is a new consideration in the context of infant MEG and it was not investigated in Larson and Taulu (3), which otherwise demonstrated the efficacy of movement compensation methodology.

Regarding the inadequacy of statistical metrics to perfectly represent the integrity of obtained source estimation results, the same is generally true for other algorithmic interpretations of data. Therefore, we strongly recommend visual inspection of data, before and after signal processing and statistical analyses. For example, if an experienced MEG researcher is unable to observe any interesting effects on visual inspection of averaged sensor-level evoked responses related to their neuroscientific question, then any subsequent statistically significant interpretations may be questionable. To prevent such problems, it is advisable to expect poor SNR in infant MEG and plan data acquisition accordingly, e.g., by over-collecting data to account for time periods when the head has moved far from the sensors.

Besides the movement distortions and other obvious infant-related MEG challenges, we have observed that cardiac artifacts are potentially especially problematic in infant MEG due to the fact that they are often very close to plausible brain signals in terms of their spatial topography. While spatial-domain suppression of these artifacts with the help of SSP or ICA tends to be efficient, there is a concern that removal of the artifact patterns could cause bias to brain signals that is difficult to compensate for. Further studies are needed to address this concern.

The above discussion relates to MEG research conducted by standard SQUID-based MEG instruments. Some of the described signal distortions may become less significant when wearable MEG systems [see, e.g., Boto et al. (31)], will be taken to use. Specifically, movement-modulated distortions should be absent in recordings conducted with sensors that are attached to the head, but movement-related artifacts still remain when the sensors are moving in the background magnetic field unless this field has been perfectly compensated for.

The main purpose of our paper was to provide information on specific challenges in infant MEG recordings that are not necessarily obvious from the experience gathered from adult MEG, and to demonstrate methodology that can be applied for robust source reconstruction results in infant MEG despite the challenges. Our recent paper on best practices of infant MEG (submitted) provides a more general and practical perspective on different aspects of a successful infant MEG study starting from paradigm planning and data acquisition while this paper contains a more detailed description of the methodology that should be useful for anyone planning to conduct infant MEG experiments.

## Limitations

As discussed throughout, one of the main difficulties of infant MEG is the mismatch between an adult-sized MEG helmet and small infant heads (due both to larger scalp-to-sensor distances and to increased space for head movement, combined with infants' tendency toward frequent motion). A general limitation is that if the head becomes too far from the sensor array (due to large head movements), the brain signal will drop below the level of sensor noise (i.e., reduced SNR). In addition, in such a situation, the ability to estimate the head position from the HPI coils deteriorates. One possible improvement would be to use infant-specific MEG hardware, such as the Artemis123 (32) or Baby MEG (33) systems, which would reduce the scalp-to-sensor distance and allow less room for movement. Obviously, this is a strategy with a multi-year implementation schedule that can only be undertaken at an institutional level. As for strategies that individual researchers might employ given their existing data collection systems, it is probably clear from the preceding sections that there is no magic bullet to fix poor SNR in an existing recording (this is equally true of adult data as of infant data). In most cases the best to be done with existing data is to meticulously apply the methods of artifact suppression described above, perhaps choosing a representative sample of the data to test a few different parameter settings of the algorithms employed, to ensure that the artifact suppression algorithms are not overly aggressive and possibly suppressing brain signal.

For collection of new data, perhaps the most practical advice is to expect poor SNR and plan task designs and recruitment efforts accordingly. Factoring in demographic controls, participants exhibiting varying degrees of uncooperative behavior, the various artifact and noise issues described here, and (for longitudinal studies) participant attrition, it would not be unheard of for the fraction of “usable” participants to be well-below 50% of the total number recruited. While not a decision to be taken lightly, sometimes throwing away data is both necessary and justified.

## Data Availability Statement

The data analyzed in this study is subject to the following licenses/restrictions: The datasets are from different studies conducted by my colleagues and these data have been made available upon request to the principal investigator, Prof. Patricia Kuhl. Requests to access these datasets should be directed to Prof. Patricia Kuhl (pkkuhl@uw.edu).

## Ethics Statement

The studies involving human participants were reviewed and approved by Human Subjects Division, University of Washington. Written informed consent to participate in this study was provided by the participants' legal guardian/next of kin.

## Author Contributions

ST contributed to the conception and design of the manuscript. MC wrote the first draft of the manuscript and created figures. EP and DM created figures and contributed to writing. EL performed statistical analysis, created figures, and contributed to writing. AB contributed to writing and design of the manuscript. All authors contributed to manuscript revision, read, and approved the submitted version.

## Funding

This work was funded by grant NIH R01-NS104585 (EL and ST), the Bezos Family Foundation (MC, EP, DM, ST, and AB), and the R. B. and Ruth H. Dunn Charitable Foundation (ST).

## Conflict of Interest

The authors declare that the research was conducted in the absence of any commercial or financial relationships that could be construed as a potential conflict of interest.

## Publisher's Note

All claims expressed in this article are solely those of the authors and do not necessarily represent those of their affiliated organizations, or those of the publisher, the editors and the reviewers. Any product that may be evaluated in this article, or claim that may be made by its manufacturer, is not guaranteed or endorsed by the publisher.
