# Supplementary figures and images for: Improving Localization Accuracy of Neural Sources by Pre-processing: Demonstration With Infant MEG Data
Source: Front Neurol. 2022 Mar 23;13:827529. doi: 10.3389/fneur.2022.827529 (PMC8983818; doi:10.3389/fneur.2022.827529)

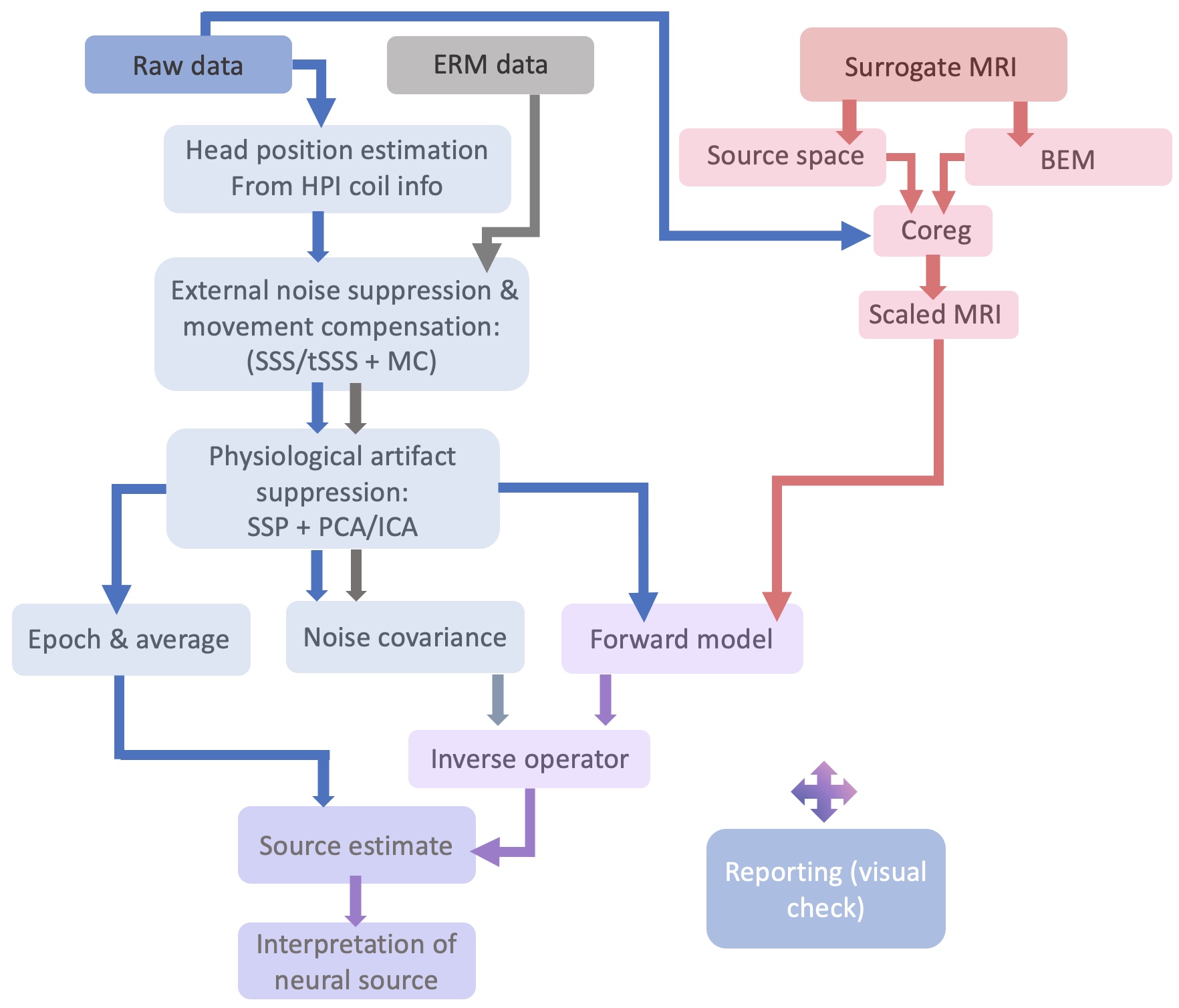

Supplement: Supplementary file 1 [file Image_1.jpeg]
